# Changes in hospitalizations due to opportunistic infections, chronic conditions and other causes among HIV patients (1989–2011). A study in a HIV unit

**DOI:** 10.7448/IAS.15.6.18089

**Published:** 2012-11-11

**Authors:** C Redondo Sanchez, G Poza Cisneros, C Galera Peñaranda, C Ruiz, A Hernandez, A Cascales, F Rodriguez, J Oliva

**Affiliations:** Hospital Universitario Virgen de la Arrixaca, HIV Unit, Department of Internal Medicine, Murcia, Spain

## Abstract

**Background:**

Reduction in mortality and morbidity in HIV patients due to the introduction of HAART have resulted in changes in patterns of hospital admissions.

**Objective:**

To examine trends of HIV patients hospital admissions.

**Design and method:**

Serial cross-sectional analysis of HIV-hospitalized patients from 1989 to 2011 in an HIV Care Unit. Each hospitalization was classified as major categories: opportunistic infections, other infections, drug-related admissions, chronic hepatopathy, AIDS and non-AIDS-related tumours and chronic medical conditions (COPD, diabetes) and as specific diagnosis: tuberculosis, PCP, CMV, bacterial pneumonia and others. We considered 4 periods of time: pre-HAART, 1989–1996; early HAART, 1997–2001; intermediate HAART, 2002–2006; and present HAART, 2007–2011.

**Results:**

We evaluated 2588 admissions. 20.7% of patients were unaware of HIV infection before first admission; this proportion did not change along the time (p=0.27). No previous outpatient follow-up was seen in 34.9% of patients. There were differences in diagnosis, mortality, age and mean inpatient stay time (Table 1) between the analyzed periods of time.

**Table:** 

	OI	HIV tumours	Non-HIV tumours	Chronic diseases	Mortality	Mean age	Mean hospital stay	Pneumonia	Resp infect	TBC	CMV	PCP	PML
Pre-HAART 682 adm.	51.7%*	5.1%*	0.8%*	3.2%*	10.1%*	36.1*	23.9*	12.1%*	14.1%*	14.1%	15%*	9.5%*	5.1%
Early HAART 632 adm.	34.5%	4%	2.2%	9%	4.6%	38.4	17.2*	21.1%	19.9%	11.7%	5%	8.2%	4.1%
Intermediate HAART 613 adm.	31.4%*	2.4%	2.8%	7.7%	4.4%	39.6	15.7	25.6%*	23.2%	11.4%	1.7%*	3.4%*	3%
Present HAART 661 adm.	21.8%*	0.8%*	4.1%*	15.9%*	3.8%*	42.9*	14.2	29.8%*	29.2%*	10.9%	1.9%*	4.2%*	2.2%

* p<0.05

**Conclusions:**

(i) HAART and older age have changed the pattern of hospital admissions with a decrease of OI-related admissions and an increase of chronic diseases and non-AIDS-related tumours and with a decrease in mortality and length of inpatient stay. (ii) Proportion of patients with unknown HIV serostatus before admission has not changed along the time. (iii) Pneumonia, respiratory tract infection and tuberculosis were the more common causes of admission.

